# Perspectives on Using Alder, Larch, and Birch Wood Species to Maintain the Increasing Particleboard Production Flow

**DOI:** 10.3390/polym16111532

**Published:** 2024-05-29

**Authors:** Roman Reh, Lubos Kristak, Pavel Kral, Tomas Pipiska, Miroslav Jopek

**Affiliations:** 1 Faculty of Wood Science and Technology, Technical University in Zvolen, T.G. Masaryka 24, 960 01 Zvolen, Slovakia; reh@tuzvo.sk; 2 Faculty of Forestry and Wood Technology, Mendel University in Brno, Zemedelska 1665/1, 613 00 Brno, Czech Republic; kral@mendelu.cz (P.K.); tpipiska@gmail.com (T.P.); 3 Faculty of Mechanical Engineering, Brno University of Technology, Technicka 2, 616 69 Brno, Czech Republic; jopek@fme.vutbr.cz

**Keywords:** wood-based panels, raw materials, lesser-known European species, alder, larch, birch, particleboard

## Abstract

Particleboard, engineered wood products as part of a large family of wood composite materials, developed in use mainly in the 1950s and 1960s to utilize inferior wood and wood waste when good-quality wood was in short supply; the annual production capacity worldwide is over 100 million m^3^. It is also necessary to have a lot of wood raw material for its production, although raw material resources are limited on our planet. In addition to the main wood species, it is therefore possible to think about the wider use of alternative, lesser-known European species of alder, larch, and birch in particleboard production. These three wood species represent an eco-friendly and sustainable wood alternative to the conventional wood raw materials used. This review confirms the diversity of the use of these three species in different fields and proves their suitability in relation to particleboard production. Fundamental research is ongoing in certain universities to determine the proportional shares of use of these tree species in particleboard (in a certain weight proportion in their core layers) for the purpose of formulating the correct technology shares and rules for their application in the wood-based panel industry.

## 1. Introduction

Particleboard, engineered wood products as part of a large family of wood composite materials, dates from the early 20th century and developed in use mainly in the 1950s and 1960s to utilize inferior wood and wood waste when good-quality wood was in short supply. The annual particleboard production capacity worldwide is over 100 million m^3^, and it is expected to reach 122 million m^3^ by 2029. In Europe, almost 30.0 million m^3^ of particleboard are produced each year, mainly for furniture applications (another particleboard volume is manufactured for construction purposes, but these waterproof particleboard are not the subject of this paper) [1,2,3,4,5].

Particleboard type P2 is suitable for dry areas. The triple-layer structure of the standard product consists of a robust core layer and two fine surface layers in order to allow clean usage. Particleboard type P2 is mainly manufactured for surface coatings. The particleboard production and its use are progressing. Due to the impact of COVID-19, several restrictions have been imposed by the world’s governments, and thus, construction and furniture activities experienced a modest downturn in 2020–2021. The conditions gradually improved after 2021, restoring the particleboard market’s growth trajectory. We are certainly aware that the significant growth in the construction of homes on account of rapid urbanization and the rising global population should represent one of the key factors fueling the particleboard market growth in following years unless there are unexpected changes, such as COVID-19 was [1,2,3,4,5]. 

Since particleboard is produced in a large volume worldwide, it is also necessary to have a lot of wood raw material from which the particleboard is made. Raw material resources are limited on our planet. Although wood raw material is renewable, its reserves are still not as high as would be desirable and there is also competition from other sectors (pulp and paper industry, combustion, and energy production) [6,7,8,9].

The question of what to produce such a large number of particleboards from is not underestimated, and worldwide research and manufacturing plants have been exploring it for several decades. Lignocellulose raw materials that do not come from trees are used, recycling of used wood has progressed significantly, and non-wood raw materials are tested. The density of particleboard is purposefully reduced by manufacturers. The lower density of particleboard means less wood raw material is needed. The lower density of particleboard may be partly to the detriment of its properties, so a fundamental reduction in density is out of the question [10,11,12,13,14,15].

Therefore, a question arises as to whether it is preferable to make more use of real wood for the production of particleboard, namely wood species that are not used at all or only used to a very limited extent and only locally for the production of particleboard. It would entail wood species of less or not at all used wood species to make a particleboard. It is the main reason for compiling this review paper [1,12].

Global research is trying to solve the possibilities of processing lesser-known and lesser-used forest trees for their use. In particleboard production, these attempts to process and correctly use lesser-known trees are limited, unfinished, and often completely absent [1,15].

It may be a matter of debate as to what are lesser-known or lesser-used trees in terms of a particular location or a certain period in terms of the available stocks of such trees in particular forests [10,16]. We will not address the representation of tree species in the forests of the world in this paper, that is, let us say, a proposal for some other paper for the authors’ collective, including forestry experts. However, some facts about the stocks of these trees in the region of Central Europe, which is mainly of concern, are obvious.

Alders are represented in the forest stands of Slovakia in the range of 1.5–2% of the total number of trees [17]. In the forests of the Czech Republic, black alder (*Alnus glutinosa* (L.) Gaertn.) is mainly represented, and overall, it is estimated that all alders can be up to 2% of the total area of forest stands [18]. The representation of alders in Austria is in the range of 2.5–3% of the total number of trees in forest stands. Even in Germany, the most widespread species is black alder and the representation of alder in the forest stands of Germany is around 3% of the total share of trees. In Poland, alders have a more significant presence, not only black alder but also gray alder (*Alnus incana* (L.) Moench). In Polish forests, alders represent about 5% of the total forest area, which makes them an important deciduous species in this country [19]. In all the mentioned countries, the presence of alder is important for biodiversity and soil stabilization in humid areas.

Data on the processed volumes of alder wood vary depending on the annual production, demand, and forestry policy of individual countries. In Slovakia, alders are not considered economically important trees, and their annual processing is around 18,000–20,000 m^3^. They are mainly used for the production of furniture and small handicraft products. In the Czech Republic, the annual processing of alder is around 35,000–38,000 m^3^. Their wood is mainly used for the production of plywood, furniture, and cladding. The annual processing of alder in Austria is around 14,000–15,000 m^3^. They are used for smaller wood products and cladding. In Germany, the annual processing of alder is estimated at approximately 40,000–50,000 m^3^. They are mainly used for the production of furniture, veneers, and smaller wooden products. In Poland, alder processing is higher, ranging between 60,000 and 70,000 m^3^ per year. They are popular for the production of furniture, plywood, and decorative elements [20,21,22].

In the forests of the Slovak Republic, birch trees represent around 2% of the total share of trees [17]. In the Czech Republic, birch makes up around 2.5–3% of the total share of trees in forests [18]. In Austria, the representation of birch is relatively small, mostly below 1% of the total number of trees in forests. In Germany, birches are mainly found in the northern parts of the country and reach a share of around 2% in forest stands. White birch (*Betula pubescens* Ehrh.) and other birch species are relatively abundant in Poland, with the representation of birches reaching approximately 7–8% of the total share of woody plants in the country [19].

The annual volume of birch processing in Slovakia is relatively low, around 14,000–15,000 m^3^. Birches are used for the production of furniture and small art products. In the Czech Republic, birch processing is around 25,000–28,000 m^3^ per year. The wood is mainly used for the production of plywood, furniture, and smaller wood products. Austria processes approximately 14,000–15,000 m^3^ of birch wood per year, primarily for the production of furniture and smaller decorative items. In Germany, approximately 35,000–40,000 m^3^ of birch wood is processed annually. Its use is similar to that in other countries: production of furniture, veneers, and decorative products. In Poland, birch processing is somewhat higher, approximately 45,000–55,000 m^3^ per year. In addition to furniture, birches are used for the production of plywood [20,22,23].

European larch (*Larix decidua* Mill.) makes up about 3.5% of the total share of woody plants in Slovakia, and its share is slightly increasing over the years [17]. In the Czech Republic, larch has a representation of around 3–4% [18]. In Austria, larch is common in mountainous areas, its share reaches approximately 5% of the total forest area, and it is an important tree for Austria. In Germany, larch has a representation of around 2–3%. In Poland, larch occurs with a share of about 3–4% within the total forest area [19].

Approximately 60,000–65,000 m^3^ of larch wood is processed in Slovakia annually. It is mainly used in the construction industry for the production of durable structural elements and cladding. In the Czech Republic, larch processing is around 80,000–85,000 m^3^ per year. Its wood is mainly used in construction, for the production of floors, cladding and durable external structures. In Austria, the annual volume of larch wood processing is around 120,000–130,000 m^3^. Austria has a strong tradition of using larch in construction due to its durability and resistance to weather conditions. In Germany, the processing of larch is approximately 130,000–140,000 m^3^ per year. It is popular in construction and furniture production due to its strength and durability. In Poland, larch is processed in a volume of approximately 95,000–100,000 m^3^ per year. Its wood is mainly used for the production of durable building structures, furniture, and cladding. These values are also qualified estimates and depend on the annual extraction and demand for larch wood [20,21,22,24].

We will assume that some wood species have greater prerequisites for processing them into particleboard, while others do not have these prerequisites [8]. In recent years, few studies have been carried out on various trees growing around the world and their possibilities for use in particleboard [25,26]. The basic question can be whether these studied trees are represented by their volume in forests in certain regions of the world in such a way that their research in terms of particleboard production is meaningful. Of course, there is no point in conducting research about a tree species that occurs, say, in a park with a representation of several individuals. Such wood species will not provide the necessary volumes of wood raw material to produce the usual large number of particleboard produced, and even if the parameters of such wood species are advantageous, for production plants such information is completely negligible. Therefore, it is necessary to know how much wood raw material from a particular tree species is available in the catchment area of a certain particleboard plant [11].

In this paper, we focused on the region of Central Europe, and we selected three types of forest tree species that are not used in Central Europe for particleboard production. These are the alder—*Alnus* spp., birch—*Betula* spp., and larch—*Larix* spp. tree species. We aimed to point out the suitability of these three trees for particleboard production from various points of view because research has already processed and published quite a diverse range of information about the possibilities of utilization and incorporation of these tree species for various purposes and some selected information from them is instructive [7,13,15].

Each of these three tree species is reviewed theoretically from the point of view of their potential for mechanical processing into chips and particles in two subsections: (1) the potential of the tree species in terms of the suitability of chipping and fragmentation to fine particles and (2) a literature overview of the previous experience of other authors in processing these three tree species into particleboard.

All this information will aid in designing new compositions of particleboard structures. We are deeply aware that the examined woods are not present in forest stands in such a way that they can be processed to 100% proportions in particleboard. Since particleboard consists of three layers, we will not plan and we do not anticipate influencing the composition of the particleboard surface layers composed of fine particles; the surface layers will remain composed exclusively of spruce species. We plan to influence the composition of the particleboard core layer and replace the part of the spruce particles with a certain proportion of one of the three investigated species. The particleboard will have an identical appearance to ordinary boards, and even a less experienced customer will not recognize the difference between 100% particleboard from spruce and our proposed particleboard with a slightly modified core layer of particleboard.

It will be a proposal for a possible and acceptable modification of the composition of the particleboard core layer, replacing part of the wood in it but replacing it with wood, real wood grown in the forest, not non-wood materials, not strange cheap non-wood materials that reduce the properties and value of the particleboard.

Our goal is to create a wood mixture for the particleboard core layer with the in-corporation certain proportion of particles from these three wood species. We choose this approach from the point of view that these three forest trees are available in Central Europe, as well as in North Europe, and they are not industrially used for particleboard purposes, given their lower quantity of available volumes. However, as suitable raw materials in a certain proportion in the core particleboard layer, they can be a recordable source of wood raw material and they can enable smooth particleboard production with a steady increase in their production volume and with a relative shortage of common wood raw materials for their production [1,2,5,27].

This review paper was prepared on the basis of a competitive and approved international research project (2022–2025) with the name Analysis of the Properties of the Less-Known European Wood Species in Composite Materials, which is financed from the research funds of two states (Slovak Republic, Czech Republic), with the participation of three research universities. This project is focused on the analysis of the properties of lesser-known wood species, primarily birch, larch, and alder, and their comparison with spruce for use in wood-based composites, especially oriented strand boards and particleboard and in the subsequent processing. We understand this project as a starting point for other international projects in broader contexts with the involvement of several countries because the lack of the majority wood species (spruce) for particleboard production is acute and, even if a minor source of wood raw material, every type is in practice, alongside other alternative raw material sources (recycled), very welcome. This research is aimed toward particleboard type P2 development with the triple-layer structure with the core layer modification and two fine surface layers conservation. However, the idea of incorporating minor sources of wood raw material into the particleboard core layer must be research-verified and sufficiently proven so that the physical and mechanical properties of the particleboard should be of the required level in terms of international technical standards and customer expectations.

## 2. Alder Species

### 2.1. Potential of Alder in Terms of Its Suitability for Chipping and Fragmentation into Fine Particles

In Section 2.1, we will try to indicate the potential of alder in terms of its suitability for chipping and fragmentation into fine particles. Our point is that the possible use and processing of alder for the purpose of particleboard should be theoretically elaborated in this review paper in such a way that the research project on our part is meaningful and that our work of a research nature is based on theoretically acquired knowledge and tested procedures published by other authors.

A genus of trees, alder belongs to the group of soft deciduous trees, and its importance in industrial processing is limited by the proportion of occurrence. Alder wood has a remarkable structure. It is a very high-demand raw material, although its mechanical processing requires more attention. Each object made of alder is unique due to the richness of the natural structures of the annual rings, bumps, and irregular course of the core in a composition with a variety of colors—from light golden, through deep, honey orange, to dark brown [28,29,30,31,32,33].

The wood of the alder is scattered porous, without a noticeable darker core. The vessels on the transverse section are not visible, while on longitudinal sections they appear as only small cracks. Fine string rays are grouped into wide bands (combined rays), on a transverse section in irregular distances and on the tangential section noticeable as long darker bands. Individual string rays are not visible, not even with a magnifying glass. The vessels are narrow and very numerous. The radial diameter of the vessels is 0.065 mm, tangential 0.05 mm, and on average, there are 100 vessels per 1 mm^2^. The length of the wood fibers is 0.30–1.01–1.65 mm, and the proportion of wood fibers is 46–58–74%, indicating good possibilities for splitting and chipping the alder wood [28,34,35,36,37,38].

An overview of the basic physical properties of alder wood is provided in Table 1. An overview of the basic mechanical properties of alder wood at a moisture content of 12% is provided in Table 2.

Published collected data on the physical and mechanical properties of alder wood, although only partially reviewed in the literature, already indicate that alder wood can be processed for the purpose of particleboard production. The individual values of the physical and mechanical properties of alder wood are basically not very different from other more commonly processed softwoods or hardwoods with a lower density similar to that of alder for the purpose of particleboard production. Therefore, alder wood seems to be applicable for our intended purpose. In Section 2.2, we will try to summarize the previous experiences of various authors in the use of alder for particleboard production and the context of alder processing.

### 2.2. Alder in Particleboard and the Context of Its Processing

The industrial production of particleboard made with the help of alder is unknown, but several research workers have published partial results of alder wood processing, either from the premise of researching its properties in chipping or gluing or directly in pressing and laboratory production of particleboard.

#### 2.2.1. Previous Experience of Particleboard Production from Alder Wood

Nemlį, G. (2003) was dealing with particleboard laboratory manufacturing from alder (*Alnus glutinosa* subsp. *Barbata*) wood. He aimed to evaluate the circumstances of the moisture content of the particles, the shelling ratio, and the effect of wood dust on the particleboard mechanical properties (static bending, modulus of elasticity, and internal bond) as well as selected physical properties (thickness swelling). His research results demonstrate that alder particles, if used for particleboard production, exceeded the EN standards for internal bond, modulus of elasticity, and static bending, although the thickness swelling values were poorer than the requirements [39].

While Nemlį, G. (2003) [39] was producing a pure 100% particleboard from alder and this is probably not entirely justified considering the not very large stocks of alder wood in forests in Europe, another research study has focused on a more sober proportion of alder in particleboards. This idea is justified, and we also prefer it. There were investigations of the wood particles (with the sieve fraction 0.4–2 mm) as a mixture of gray alder (*Alnus incana* (L.) Moench) and the lignin powder with a concentration of 15 or 25%. Their thickness swelling, density, water absorption, internal bond strength, modulus of rupture, and modulus of elasticity in bending were determined. It was detected that the amount and the source of the added lignin clearly affected all the tested particleboard properties [40]. It seems to us from this that alder wood is acceptable for the purpose of particleboard production.

Kumaş et al. [41] conducted research on the effects of the drying temperature of the particles and the pressing temperature on particleboard’s technological properties, which were produced from black alder wood. Wood particles with different drying temperatures (120 and 150 °C) and pressing temperatures (150 and 200 °C) were used. If the particle-drying temperature was higher, the pressing temperature had an absolute effect on the particleboard’s dimensional stability. An increased pressing temperature improved the board’s mechanical properties. This research needs to be appreciated and taken into account, as the board’s mechanical properties are important and the tried and tested path to achieve them must be followed.

In their research, other scientific workers dealt with the assumptions of alder processing from various points of view, and they came to conclusions and findings that are helpful in the investigation of this wood as a suitable raw material for partially covering the needs of wood raw material to produce particleboard.

#### 2.2.2. Analysis of the Chemical Composition of Alder Wood as a Factor in Particleboard Production

It is very important to know the chemical composition of alder wood in detail because the individual components can partially affect its processing and the future particleboard properties from different aspects.

Geffert at al. [42] evaluated the chemical components of alder wood (*Alnus glutinosa* L.) and also investigated the changes in the chemical components caused by steaming with saturated steam at three temperatures—105 °C, 125 °C and 135 °C. They reported that cellulose is present in regular alder wood at 44.1%, polysaccharides at 77.2%, lignin at 22.0% and extractives at 6.6%. The greatest changes in the alder wood after steaming were observed in the polysaccharides content, with the greatest decrease at a temperature of 135 °C. The cellulose content was relatively stable and small changes was caused by the degradation of the more unstable wood components were apparent. Alder contains a relatively wide range of different substances, including various polyphenols, diarylheptanoids, flavonoids, triterpenoids and steroids. Black alder and gray alder contain the triterpenoids alnusenone, taraxerol and taraxerone. The steroids brassinolide and castasterone have been isolated from sticky alder. Among the biologically active components of alder, diarylheptanoids are the most important. Phenolic compounds and flavonoids are carriers of the antioxidant effect. The yellow dye kaempferol is contained in the bark of *Alnus nitida*.

Alder wood contains extractive substances affecting its gluing process. Janceva et al. [43] studied the possibility of using condensed tannins-rich extracts from gray alder (*Alnus incana*) as well as from black alder (*Alnus glutinosa*) bark in the production of particleboard and plywood adhesives. The extracted microparticles from alder bark residues used into the composition of the adhesive system showed positive potential for application as a filler. They certainly play a significant role in particleboard production.

A seemingly less related study was published by Öztürk et al. [44], but the results of the studied alder in it, among other tree species, are very important. The waste-expanded polystyrene particles of five different densities were used in the production of composite particleboard. Half of the alder or other species chips were dried in a drying oven and the other half just naturally conditioned at room temperature, and then a three-layer composite particleboard 18 mm thick was produced. The thermal insulation performance of the composite particleboard panels increased with the use of waste-expanded polystyrene foams with a density of 30 kg/m^3^, according to the analysis findings. The lowest thermal conductivity values were acquired from the expanded polystyrene waste foams with the low density of 13, 18, and 22 kg/m^3^ for the panels produced with alder, pine, and poplar in the natural-drying condition. Technical drying showed better thermal performance than natural drying. The accompanying substances in alder wood have been identified, as in previous research [43], and need to be considered.

In their previous study, in comparison with [44], Öztürk et al. [45] dealt with the thermal conductivity of wood material, which is superior to other building materials because of its substantial porous structure. Their efforts aimed to produce a relatively new wood composite material with insulating properties by using insulating material polystyrene instead of formaldehyde-based adhesives as the bonding material. Five wood species (alder, beech, poplar, pine, spruce) were used and three-layer particleboard with the thickness of 18 mm was produced in this study. The thermal conductivity values obtained from natural drying were found to be higher than from technical drying, according to the results of the study. Alder wood was fully operational in this trial, and it was recommended for further research.

#### 2.2.3. Investigations of Alder Wood Particle Storage Methods

For our needs, it seems that the research by Szadkowska et al. [46] is very stimulating. Their study was focused on investigating the effect of the storing methods for wood particles from two species, alder (*Alnus* Mill.), and pine (*Pinus sylvestris* L.), on the basic chemical composition. In the study, two storing methods for woody biomass were used: an open pile and a covered pile. The wood was cut down at the end of November and it was freely laid as industrial rough chips for 8 months from December onwards. After this time had passed, the material was collected again for its chemical composition analyses as well as the enzymatic hydrolysis. The influence of the type of storage on the composition of the individual structural components of the wood was demonstrated as the results of the chemical composition analysis of the wood for both studied species.

On the basis of the results of the chemical determinations carried out on the wood biomass of alder wood stored in an open pile and covered pile for 8 months, it can be concluded that the wood storage method plays a significant role in the enzymatic hydrolysis efficiency, which has been statistically confirmed. High yields of enzymatic hydrolysis were obtained for alder wood biomass being stored in an open pile; moreover, the alder biomass had a higher yield of enzymatic hydrolysis when using dyadic enzymes than biomass from softwoods. A higher cellulose content (+3.6%) was observed when the alder raw material was stored in a covered pile. The holocellulose content had a similar level for alder raw material storage, being 70.1% and 71.9% for an open and covered pile, respectively. The hydrolysis effectiveness increased with the time as well, independent of the material type that was hydrolyzed. Thus, it is obvious that raw alder wood (like other types of wood) stored for too long is not suitable for the production of quality particleboard.

#### 2.2.4. Consequences of the Thermal Treatment of Alder Wood for Particleboard Production

The effect of elevated temperatures on alder wood quality as studied in the past by other authors is important in terms of determining the correct pressing temperature for particleboard using alder wood. Thermal treatment of alder wood has been investigated by Lee et al. [47]. They wanted to offer several advantages to the treated wood, such as exclusion of oxygen during the treatment process and even more and the rapid transfer of heat into the wood samples. Alder wood treated in soya oil at 180 °C and 200 °C for 6 h and 10 h exhibited superior resistance against *Postia placenta*. Urea formaldehyde-bonded particleboard was soaked in palm oil for 24 h before being thermally treated in the oven at 180 °C, 200 °C, and 220 °C. Such temperatures did not destroy the alder wood and pressing it into a particleboard at the level of 230–240 °C, or even slightly higher, is feasible.

The color changes and decay resistance against white rot fungus (*Pycnoporus sanguineus*) of the samples after thermal treatment were investigated. After thermal treatment, the lightness of the samples decreased, and the extent of the darkening increased along with an increasing treatment temperature. With the expected lamination of the particleboard surface, this argument will not matter, but when using raw particleboards without surface treatments, it is necessary to draw attention to this.

#### 2.2.5. Comparisons of Selected Alder Wood Resources from Various Stem Parts for Particleboard Production

The selection of a specific stem part of alder wood resources for particleboard production is not possible during production. Nevertheless, it is good to know that the use of an insufficient general wood mixture in particleboard production can affect the properties, as further research has pointed out. Alder was also the subject of the project on binderless bark particleboard made from bark waste obtained from gelam (*Melaleuca viridiflora* Sol. ex Gaertn.) [48]. The particleboard’s thermal insulation properties were determined as well. Up to four different temperatures (140 °C, 160 °C, 180 °C, and 200 °C) were set up to make single-layer binderless bark particleboard with a density of less than or equal to 590 kg/m^3^. The results showed that the pressing temperature affected the physical and mechanical properties of the manufactured boards. As the pressing temperature was increased from 140 °C to 200 °C, the average values of the mechanical properties also increased, even though the increase was not significant for the MoR and MoE values at pressing temperatures of 180 °C and 200 °C. The best physical and mechanical properties were obtained from the particleboard pressed at a temperature of 200 °C, with an MoR value of 3.97 N/mm^2^, MoE value of 758.05 N/mm^2^, internal bonding (IB) value of 0.6 MPa, TS24h value of 4.94%, and WA24h value of 16.3%. For us, this implies that higher pressing temperatures for the particleboard are fine even if the pressed chip mixture is diverse.

The characteristics of particles made of straw of selected grain species, including comparisons with alder particleboard purposed to produce lignocellulose particleboard, were tested by Dukarska et al. [49]. Their research offered the characteristics of three different species of grain straw particles applied in lignocellulose particleboard production. The research aimed to determine the physical properties of particles of less traditionally input raw materials and to evaluate their usefulness in the process of manufacturing lignocellulose particleboards purposed for building constructions and to measure the particles’ usefulness in the process of the lignocellulose particleboards manufacturing oriented for the building constructions. Every investigated material was characterized by the different tapped bulk density, varied angle of repose, geometry, poured, and the slippery angle of repose. The material produced was sorted using screens with a mesh diameter of 4 mm and 2 mm in order to select the material for the surface layers and the core layer. The desired fraction for the core layer consisted of particles retained on a sieve with a mesh size of 2 mm. Particles larger than 4 mm were reground and sorted, and particles smaller than 2 mm were used for the surface layers. These values need to be known and evaluated, and this is also the case with our intended exploration of less traditional trees, which has been confirmed by this research [49]. Alder wood has not been primarily researched, but references to it and its connections are also applicable to the processing of alder wood into particles. The paper’s conclusion, and for us a lesson, is that each material is characterized by different geometry, poured, and tapped bulk density, varied angles of repose, and slippery angles of repose. Differences in the sizes and shapes of particles of various grain species and in their moisture content strongly affect the properties of particleboard produced with their use. We must be aware that difficulties in storage, transport, and particleboard production may happen.

Salca [50] examined black alder species (*Alnus glutinosa* L.) from various points of view as a resource for value-added products. She assumed that due to its workability, properties, and appearance, black alder can be considered a suitable particleboard raw material. Her review offers a contour of the potential of black alder as a wood raw material. She emphasized the application and alder wood properties in terms of light exposure and the color changes under air, the surface quality of processed particleboard during sanding and milling, coating properties, and the process optimization. The findings of her study help with the practical applications so that this woody species can be processed and utilized more efficiently for value-added products. The average bending strength values found for alder wood were in the range of 44–110 MPa. Black alder as a less-utilized woody species has been shown to have considerable potential for industry so that its use can successfully reduce the import activities of other woody species.

#### 2.2.6. Possibilities of Chemical Modifications of Alder Wood for the Purpose of Improving the Quality of Particleboard Production

As in Section 2.2.4, this subsection summarizes the chemical properties of alder wood and their potential impact on particleboard production. The synergistic influence of the intumescent coating that contains titanium dioxide and antimony trioxide onto alder and spruce woody species was brought to the attention of a team of researchers led by Torun et al. [51]. It is good to know the reactions of alder wood with various chemicals. The results confirmed that the wood surfaces coated by different percentages of ammonium trioxide and titanium dioxide added to paint had a positive influence on the wood’s physical properties as a promising raw material for particleboard production with a certain share of alder particles.

Lovaglio et al. [52] tested the wetting behavior of alder wood surface (*Alnus cordata* (Loisel) Duby) from the point of view of the influence of alkyl ketene dimer and the thermo-treatment before and after a current artificial weathering test. The FTIR analysis carried out by them promoted their hypothesis that the alkyl ketene dimer could make the bonds relatively chemically stable, even when using a rather small concentration. Their findings provide important information for any future research and for alkyl ketene dimer utilization on lesser-used wood species.

#### 2.2.7. Use of Alder Wood for the Production of Other Types of Wood Composites and Other Context

Alder wood plywood has been produced for many years and has been studied in detail. The context of gluing alder veneers is fully applicable to gluing alder chips, since in the vast majority of cases it will involve the use of an identical type of glue. Bekhta et al. [53] dealt with the properties of plywood composed of thermally densified and non-densified birch and alder veneers. This, in turn, is another view of the possible behavior of alder wood, applicable to its intended processing into particleboard. Their observation aimed to develop plywood with two wood species (black alder and birch) and two veneer treatments (thermally densified and non-densified) to evaluate the influence of various lay-up schemes on the plywood’s properties. The results demonstrated that the wood species, type of construction, and thermal densification of the veneer applied influenced the examined mechanical and physical properties. Black alder veneers can be placed into the inner layers of plywood without the danger of reducing their shear strength. An increase in the share of the thermally densified veneer in one panel leads to the higher density, shear strength, and bending strength of the plywood. It was also demonstrated that non-treated alder veneer, despite exhibiting lower strength properties than birch veneer, could be used with a proper lay-up scheme in the plywood-based product industry. Therefore, mixed-species plywood enables the increased utilization of low-grade, low-density, and low-cost alder wood veneers as inner veneers in panels to reduce the production costs and increase the mechanical properties of predominately low-density alder wood plywood. This is a good insight when further reflecting on the processing of alder wood.

In a broader context, these issues are also dealt with in a book compiled by the editors Bekhta and Krystofiak [54].

Alder plywood is also of interest to other researchers. Öncel et al. [55] aimed to determine both the tensile–shear strengths of plywood produced from Uludağ fir (*Abies nordmanniana* subsp. *bornmülleriana* Mattf.), alder (*Alnus glutinosa* L.), Scots pine (*Pinus sylvestris* L.) and Samsun poplar (77/51 *Populus deltoides* Bartr.) by using phenol formaldehyde resin. They tested the rotary cut veneers in combinations of poplar–fir, poplar–pine, poplar–alder, and poplar wood, along with the effect of wood types on the adhesion glue quality. As a result, the tensile–shear strength values of alder–poplar plywood were found to be average. It was found as well that there was not a significant difference between the alder–poplar and fir–poplar plywood types regarding the tensile–shear strength. All the plywood types tested in this study, including alder plywood, are of suitable quality for outdoor use. Another research project went even further. The authors continued the research on laminated veneer lumber (LVL) from alder wood, the properties of which also passed the standard values. Since alder wood has passed these tests, it will certainly behave appropriately, while observing all the necessary technological conditions, including in the context of its gluing in combination with spruce in the core layers of particleboard.

An extension of the idea of this research was reported by İlkay and Mengeloğ [56]. They verified the layer combination effect on the equilibrium moisture content, oven-dry specific gravity, water absorption and thickness swelling, modulus of rupture, modulus of elasticity tensile–shear strength, and compression strength parallel to the grain of LVL manufactured from alder and poplar. Urea-formaldehyde resin was used in LVL production. LVL was produced in 10 different combinations. The highest compression strength, MOR, MOE, and oven-dry specific gravity values were obtained with alder veneers used in overall plies. But, it is interesting that the highest tensile–shear strength values were obtained with poplar veneers used in overall plies. With the increased use of poplar veneer in LVL production, an increase in the amount of water absorption and thickness swelling was observed. It was noticed as well that as the contribution rate of the alder veneer in lamination increases, the modulus of rupture, modulus of elasticity, oven dry specific gravity, and compression strength values were raised. Also, based on this study, alder is a suitable wood for its mechanical processing and use in composite materials. 

Suvorova and Romanenko [57] tried to prove that it was possible to obtain wood with high-performance properties based on mechanical and chemical action because of the optimization of technological processes and use of temperature exposure. The initial raw material was hardwood (alder, aspen), which is not too commonly used in construction and in the production of finished materials. The condition for obtaining wood with high operating properties (increasing strength, density, reduction of water saturation, ensuring the samples dimensional stability for a long time) is the ability of wood as a natural polymer to change properties under the combined effect of pressure and temperature.

Romaneko himself supported these activities in another published paper [57].

It is a very correct aspect when we also evaluate the possibility of dust formation during alder processing. This view of alder was dealt with by Pędzik et al. [58]. Wood dust poses the risk of explosion and fire and a threat to the health of employees, worsens the quality of processing, accelerates the wear of machines, and requires large financial outlays for its removal. This study is focused on research on the extent to which the grit size of the sanding paper affects the size of the wood dust elements and the share of the finest elements that, when dispersed in room air, may form the respirable fraction. Six species of hardwood (alder, beech, ash, oak, walnut, and hornbeam) and three species of softwood (larch, spruce, and pine) were used in this research. Based on the results obtained, they found that the dust elements’ sizes were also obtained in the case of alder, which means that we must be careful when processing it.

The dust generated during the processing of alder was also investigated by Sydor et al., and they achieved similar results, with an emphasis on dust arising from alder wood [59].

If we look at the issue of alder processing from the perspective of the future, we must also be aware of the issue of disposal of alder products at the end of their useful life. Qiu et al. [60] were active in this regard. The paper deals with the thermal decomposition and the behavior in combustion of two kinds of plywood used in composite floorings. The research study was prepared on the use of a fire propagation apparatus. The combustion parameters, including the heat release rate, ignition time, effective heat of combustion, and mass loss rate, were analyzed. The ignition risk and heat release of alder plywood exist. It follows that the disposal of alder products by incineration is not an operationally suitable activity, as it will be more appropriate to extend their life through recycling and circular economy.

The paper by Sedliacikova et al. [61] deals with the issue of the color tones of wooden and furniture products. This issue can be interesting from the point of view of particle color changes in the composition of particleboard, not so much from the point of view of the real color but from the point of view of chemical changes in wood that must be perfectly bonded together in the form of particles within the particle-pressing process. The main aim of their study was to identify the supply of the color tones of wooden and furniture products and to map the interest in these wood color tones for potential customers in Slovakia. It is necessary to increase the supply of wooden and furniture products with the natural color of the wood and at the same time in the color tones of white, gray, and brown. The current demand for modified alder wood and thermowood is significantly higher than the supply of such products on the market. The results of this study represent an opportunity for woodworking and furniture factories to adapt their product range according to the needs of potential customers, which will bring them a higher turnover and help to overcome the possible problems. Also, based on this study, we can conclude that alder wood can also be processed into the particleboard core layer from the point of view of the supply of wood products with the natural color of the wood.

#### 2.2.8. Overall Evaluation of the Context of Alder Wood’s Possibilities in Particleboard Production

It is clear from the mentioned and other unmentioned literature sources that alder has suitable prerequisites for its processing into particleboard. The sticky alder (*Alnus glutinosa* (L.) Gaertn.) is a deciduous tree, but it is one of the softest woods, which is not very common in the category of deciduous trees. This could be interesting for the purpose of our project, as the low density of particleboard and OSB boards is currently in high demand all over the world. Another characteristic of alder wood is its resistance to impacts, but also its low stiffness. Alder wood has a high resistance to exposure in outdoor conditions in humid or even drier places; there is a high probability of creating few, if any, cracks.

The durability of alder is mediocre in the range of hardwoods. It is impossible to expect the quality of alder wood to be comparable to oak or maple. Alder wood can absorb moisture, which can affect the quality of particleboard and OSB boards. Alder wood is very suitable for any manual processing, and it also polishes well and accepts all kinds of colors. It is suitable for making models of various products. Winter harvesting of alder and its quick cutting is recommended, and the protection of the ends of the logs is also suitable. Lumber must be stored in airy cages; thin lintels should be used. The use of alder wood for furniture has been known for a long time. The appearance and, usually, the lower price of alder wood are chosen by many people. In addition, its durability also makes alder home furniture long-lasting. Alder is used to make chairs, tables, cabinets, kitchen furniture, and other types of furniture. Alder wood is known for the production of interior doors thanks to its uniform structure with prominent knots. Alder is a relatively soft wood, which makes it easier to repair those places where damage has occurred. It is not suitable for the production of exterior doors; it may not be sufficiently resistant to the fluctuations of harsher weather. 

The quality of alder wood may not be sufficient for high-quality windowsill boards or shaped wooden products. Alder wood is extremely suitable for decorative purposes in the form of covering decorative veneers (or even micro-veneers) for large-area panels for wall coverings. Its distinctive pattern and regular structure lend the slatted panels a beautiful appearance. Stabilization of alder veneers with a durable transparent coating system is necessary. Due to its softness, alder wood is suitable for turning, milling, and the production of various noble objects. It is also suitable for carving purposes; alder products are attractive. Alder is one of the most suitable types of wood for the production of kitchen utensils.

Alder plywood is well-known and of high quality. Alder is easily processed by centric peeling technology; the manufactured structural veneers are suitable for the surface or middle layers of plywood. The insufficient strength or softness of alder for some applications can be eliminated by making plywood from it. Alder plywood is used in the construction of doors, furniture, and the like. Some musical instruments, such as guitars, are made from alder. The guitar body is characterized by a robust and clean sound, a balanced entire sound spectrum, and other suitable acoustic properties. Alder wood is only used to a limited extent for pulp and paper production [28].

It can be concluded, based on this information from the published literature, that alder wood has the potential to be pressed with spruce particle mixes into the core layer of particleboard.

## 3. Birch Species

### 3.1. Potential of Birch in Terms of Its Suitability for Chipping and Fragmentation into Fine Particles

Even in the case of birch wood, there is a collection of available data on the potential of birch in terms of its suitability for chipping and fragmentation into fine particles. Birch is an appropriately more analyzed tree in the literature context, because its use in the veneer-based and plywood industry is so decisive that birch wood research is more extensive and long-term. The literature also contains data on the possible uses and processing of birch wood for the purpose of particleboard. Therefore, in this subsection of the review paper, birch wood is theoretically elaborated, which includes utilizable meaningful works of a research nature. These laboratory works must include theoretically acquired knowledge and birch wood-processing procedures published by other authors.

Birch (*Betula verrucosa* Ehrh.) and other birch species belong to the group of hardwoods, and its importance in industrial processing is limited by the proportion of occurrence. It is a very important tree species for the north of Europe and the experience of using it to produce particleboard is greater than in the case of alder [28,33,53]. The color of birch wood is white with a hint of red. The boundaries of the annual rings are indistinct. It creates a very nice texture on a tangential cut, especially wood with sleeping eyes or flaming. Birch is a sapwood tree species; the sapwood is not visible to the naked eye. It is a scattered porous wood. The width of the annual rings ranges from 1.2 to 2.0 mm. We can only see the shaft rays in birch on a radial section [28,33].

Of the total volume of wood, wood fibers make up 65.8–75.7%, vessels 10.6–21.4%, stem rays 10.8–11.7%, and wood parenchyma 2%. This is scattered (diffuse) in birch wood, i.e., the cells are scattered evenly over the annual rings. Spindle-shaped parenchyma cells occur in birch wood. The diameter of the vessels is 0.02–0.1 mm. The thickness of the wall is very small at 1 μm. The perforation of birch vessels is always stepped. The colons do not have a torus. Birch has a vascular tracheid, the length of which does not exceed 0.5 mm. The width of the libriform fiber in birch is 0.219 mm, and the thickness of the cell wall of the libriform fiber is approximately 5 μm. All the microscopic parameters indicate good possibilities for splitting and chipping the birch wood. Birch wood belongs to the medium–heavy woods, and it is slightly hard. It impregnates and stains well [28,34,35,36,37,38].

An overview of the basic physical properties of birch wood is provided in Table 3. An overview of the basic mechanical properties of birch wood at a moisture content of 12% is provided in Table 4.

Birch, in terms of the air durability, can be classified among the low-lasting wood species. In terms of durability in contact with the ground, where there is a risk of damage by all types of rot, it is classified as a non-perennial woody species (Table 5).

The values of the physical and mechanical properties of birch wood collected from the literature are much more comprehensive than the information collected for alder wood, as listed in Section 2.1. It follows that the processing of birch wood into particleboard will be more refined and less experimental. Birch wood is well-studied in many, especially Nordic, countries, and this literature review is proof of that. The values of the physical and mechanical properties of birch wood are very favorable for the production of structural and decorative veneers, and the literature research shows that they are favorable for chipping wood and the production of various chips. Therefore, it is assumed that birch wood will also be suitable for the production of specific chips needed for the purpose of particleboard production. Section 3.2 provides a summary of the previous experience of various authors in the use of birch wood for particleboard purposes and in the context of birch processing and its possible impact on the spruce and alder particles mixture.

### 3.2. Birch in Particleboard and the Context of Its Processing

Kollmann et al. [65] and Maloney [66] drew attention to the great importance of the quality of wood as an input raw material for the production of particleboard in the 1970s. In their well-known publications, they emphasized the importance of the technical parameters of the particles used for particleboard production (dimensions, thickness, moisture content), and they also emphasized the suitability of certain types of wood species in terms of their structure. They stated that it is impossible to produce high-quality boards from inferior raw materials. From the range of available woods, in addition to conifers, they also recommend hardwoods with a lower density, and birch (as well as alder) meets their requirements.

#### 3.2.1. Previous Experience of Particleboard Production from Birch Wood

Varis [67] focused on Nordic wood processing and particleboard production in Finland, mainly from spruce but also from birch, which is a reality. The main raw material in Finland is small-diameter spruce timber from thinning, sawdust generated in sawmilling, and particles and veneer trash created in the plywood industry. It is trash from the plywood industry that is mainly the source of birch particles, in addition to spruce particles, in the production of particleboard. Birch particles are a little heavier, but they are suitable for the quality of Finnish particleboard.

Varis [67] noted that in Europe, it is more difficult with raw materials for the production of particleboard, because in addition to a larger assortment of tree species, including birch, recycled wood and wood from demolitions are used, but the research also coped with such raw materials, as long as they do not exceed a certain reasonable share in particleboard.

The importance of suitable input raw materials for the production of particleboard is emphasized in the publication by Irle et al. [12] and, with an emphasis on Central and Eastern Europe, in a compiled publication by Reh [68].

Varis provided up-to-date information usable in pressing birch particles mixed with spruce, but also older research was performed by Liiri [69] is impressive and incorporating. Three-layer interior particleboards, prepared from birch, alder, pine, spruce, and aspen, were tested for their strength properties and also their water absorption and dimensional stability during soaking or variations in relative humidity. The wood density proved to be the main factor in determining the suitability of the raw material, affecting both the strength and dimensional stability. The bark in the particles for the middle layer did not adversely affect the board’s properties. All the tree species appeared to be suitable for particleboards. Birch provides the boards with a density of more than 800 kg/m^3^, which is already an unacceptably high density from today’s point of view, but it tells us about the suitability of birch (and alder) for making particleboard. However, it could be satisfactorily mixed with lighter wood species.

Good news for processing birch into particleboard also comes from the research by Baharoğlu et al. [70]. They did not deal directly with specific tree species but compiled a very decent overview of the technological matters necessary to produce high-quality particleboard from alternative raw materials. They analyzed a variety of literature sources, and they summarized that the main factors that determine the potential of alternative raw materials to be used in composites production are their physical and chemical properties, the possibility of seasonal storage, local availability, the supply volume, and their carbon neutrality. Their study aimed to review the current state of knowledge on the possibilities of the use of various raw materials in the production of particleboard. Based on this review, an experiment was performed to identify the raw materials with the highest potential, considering their quality and the availability of boards made from them. Suggestions were made as well regarding the search for new raw materials, based on their production potential. Their summaries clearly show that some raw materials, even non-wood, may be acceptable for particleboard production, but real wood is the most suitable. 

Similarly, Krug et al. [71] considered and accumulated technical information on particleboard that is presented as an important product. They also stated that particleboard, if it is to be of good quality and meet all customer requirements, must be made from the right raw materials and with the right manufacturing process. Real wood is an irreplaceable raw material.

Particles made from birch were researched by Çamlıbel and Aydın [72]. They closely watched the effects of a continuous pressing speed (580 or 600 mm/s) and the time of the conditioning (without and 72 h) on selected physical and mechanical properties and the formaldehyde content in particleboard. They based their research on the use of birch elements in MDF boards and particleboard using UF glue mixtures, where birch has proven itself well.

Skurydin and Skurydina [73] summarized the results of the digital differential spectrometry application in the processing of the results of the study of the structure of wood and wood-based materials by dynamic mechanical analysis (DMA). The results of the application of the DMA method and digital data-processing are presented in the study of the dynamic mechanical characteristics of wood of birch and apple, a composite made of hydrolyzed wood, as well as particleboard. It is proven that the use of digital processing of experimental data provides accurate results, simplifies their interpretation, as well as expands the understanding of the processes occurring in wood-based materials at the level of the intermolecular interaction. According to this method, birch turned out to be a suitable wood for particleboard manufacturing.

Singh et al., in their study, discussed birch and aspen used for the development of modern engineered wood [74]. Wooden elements used for the development of engineered wood have various factors in terms of the shape, size, surface conditions, density, and other dimensional parameters that affect the mechanical as well as physical properties of the final product. Also, the permeability, pH, element shape, and aspect ratio play a vital role in the manufacturing process for engineered wood. A wide range of compositions and percentages of raw elements and resins could be used to obtain different results in terms of the physical and mechanical properties as per the application of particleboard. The addition of selected suitable resins in small quantities along with the resin is also recommended as it upholds the mixing process by assisting in achieving homogeneity. Birch stands out as a suitable material comparable to well-known raw materials used to produce particleboard.

Razinkov and Ishchenko [75] looked at the issue from the perspective of the shortcoming of particleboard for the production of cabinet furniture. This is related to the properties, which must meet the standards even though particleboard-produced volumes are increased. Important disadvantages of particleboard include the toxicity associated with the release of formaldehyde, rather low mechanical (strength) properties, and limited areas of application of particleboard (mainly indoor). They watched the dynamics of the technical requirements for the properties of particleboard according to the standards and they looked for the reason for the reduction in the requirements concerning the strength of particleboard in bending and, in this regard, the constraint of the application areas of particleboard. Their research included the type of wood species used for particleboard production (mainly birch, alder, pine, aspen, and some others), and birch comes out positively from this research.

#### 3.2.2. Possibilities of Particleboard Production from Birch Wood and Alternative Adhesives

Since birch is a very important wood species for the production of plywood for Northern Europe, quite a lot of scientific works deal with the processing and use of birch wood or birch bark in terms of processing it with cutting tools. This is interesting for any cutting tool operation, which can also be chipping machines. Tupciauskas et al. [76] investigated residual birch veneer shorts and the birch outer bark that are among the main residues from birch plywood production. They have a strong potential to be converted into value-added products. Birch (*Betula pendula*) outer bark contains up to 45% (on the dry basis) suberin and it has been recognized as a promising resource of suberinic acids; their bonding potential has been proven. The study evaluated the particleboard production made of birch wood particles obtained from residual veneer short pieces and the adhesive enriched with birch bark extracts, and it indicates to us that a mixture of birch wood, even in a partially inhomogeneous mixture, with the possible presence of glue, is acceptable in a reasonably small amount for particleboard core layer production.

Jeżo and Wronka [77] focused on post-extraction birch bark residues as a potential binder in particleboards. Their paper also concerned the possibility of using post-extraction residues obtained during the extraction of suberinic acid as a formaldehyde-free and ecological binder in the production of particleboards. Their research aimed to address the following issue: since suberinic acid itself is a good binder in the production of particle boards, it is worth checking whether the post-extraction residues also have similarly good properties in joining particles in particleboards, depending on the size of the wood particles. In addition, the use of post-extraction residues of bark, and thus the elimination of synthetic adhesives in the wood-based composites production process, allows the reuse of wood raw material, which fits perfectly with the idea of upcycling. The tests showed that using post-extraction residues of birch bark, using 10% and 20% resination, the requirements of the EN 312: 2010 standard [78] were met in the case of the modulus of elasticity for boards made of the largest wood particles used in the tests. The resination and the size of wood particles contributed to the improvement of the properties of the tested boards.

This topic also caught the attention of Makars et al. [79], and they dealt with the investigation of the furfural formation and mechanical properties of suberinic acid-bonded particleboard depending on the parameter preparation. The adhesive included the silver birch (*Betula pendula*) outer bark suberinic acidic, which can catalyze furfural formation from xylans in wood particles that are used for particleboard preparation. As part of their study, the impacts on the technological parameters (wet adhesive pH) of glycerol as an additive to the adhesive and the hot-pressing temperature were investigated in particleboard. The mechanical properties (modulus of elasticity, bending strength, and thickness swelling) of particleboard were studied.

Makars et al. [80], in their subsequent publication, expanded on this idea and the research on birch (*Betula* spp.). They also discussed the outer bark from the point of view of betulin as a valuable product. They realized that after the removal of betulin extractives, suberin-containing tissues were left.

#### 3.2.3. Use of Birch Wood for the Production of Other Types of Wood Composites

It has already been stated several times that birch plywood and other birch products are essential for the Nordic countries. Hence, a substantial part of the published scientific information is applicable to the theory of birch particle production and their pressing. Tupciauskas et al. [81] tried to confine, replace, or maybe even eliminate the synthetic resins from wood-based composites because expanding the range of raw lignocellulosic is still important and attractive. Many pretreatments of lignocellulosic have been carried out, among which steam explosion resulted in the superior physical and mechanical properties of the obtained binder-less particleboard. Emissions of volatile organic compounds were investigated in the framework of the study from binderless particleboard obtained from different raw lignocellulosic and birch suberinic acid-bonded particleboard. The results showed that the number of detected volatile organic compounds and their chromatographic peak area varied significantly depending on the raw lignocellulosic, board density, and post-treatment (overlayering), decreasing over time.

The surface properties, like the roughness, color changes, hardness, wetting, and density, of heat-treated silver birch (*Betula pendula*) veneers were studied by Dudik et al. [82]. None of the parameters studied has proved the negative changes due to the temperature adjustment. Both in terms of the properties and in terms of the valuation, there is considerable potential for birch to replace, for example, beech, especially in the furniture industry, by application in the form of heat-treated veneers after the required heat treatment.

Kowaluk and Jeżo [83] investigated the modulus of elasticity and the contractual compression strength when compressing (MOEC) three different wood-based composites with several structure types with birch wood elements (veneers, particles). The density profiles and density shares of the samples were analyzed as well, and all of the examined wood composites exhibited a U-shaped density profile. The study results pointed out that there is no significant correlation between the density and the obtained parameters under compression. Observation of density share led the authors to conclude conversely than the results suggested. Therefore, the key factor effecting the compression performance of the samples was the adhesive area and solid glue content within the wood composites. It can be assumed that the bigger the total contact wood particles surface coated with adhesive resin (and thus the sum of the effective surfaces of the adhesive joint), the better the mechanical properties of the wood composites.

#### 3.2.4. Other Birch Biomass for Particleboard Production

Dumitrascu et al. [84] considered three different fast-growing wood species, namely birch, poplar, and willow, in their research. They drew attention to the fact that the referred to species may have certain potential to replace the softwoods or wood species mixtures frequently used in the production of OSB (oriented strand boards). Their study described selected mechanical and physical properties of the wood species used that influence the OSBs’ performance when made using 100% strands from each wood species. The strands of wood were first cut, then dried, screened, and sorted to form the middle and surface layers of the OSBs. The strands were blended with pMDI adhesive (polymeric diphenyl methane diisocyanate) and compressed with a heated hydraulic press. OSBs made of birch presented excellent elastic properties. The results of the experimental study can have industrial applications for the efficient utilization of less used and less known raw material or, as in our case, for the use of birch particles mixed with spruce particles in the particleboard core layer.

Xu [85] thought in 1994 that a huge number of material and processing variables influence the properties of particleboard. He thought that not too much is known about the particleboard’s internal structure, and an essential principle or theory interrelating the structure, processing, and properties has yet to be developed. Basic knowledge of the particleboard structure is necessary to fully understand the present particleboard technology, but it is also important for the future upgrading and development of wood-based composites. The study was elaborated to develop some of this knowledge base. The two major objectives of this study were (1) to understand the influence of raw material characteristics on the horizontal density distribution, and (2) to specify the effect of particleboard nonuniformity defined by the horizontal density distribution on selected properties. He used birch particles in the research, and with the help of them, he determined 26 particleboard panels. All of them were made with precisely cut particles to study the first objective, while 30 particleboards involving different element sizes and distributions, different wood species and combinations were prepared to study the second objective. With the use of relatively larger specimen sizes, particleboard made with larger particles exhibited more substantial density variation, while particleboard made with smaller particles showed larger variations at smaller specimen sizes. Two aspects of lacks, namely the size and number, were identified as factors contributing to the relation between the particle size and horizontal the density distribution.

Carre [86] analyzed forest biomass for the manufacture of particleboard. He used Belgian samples of the proportion of aerial biomass greater than 7 cm in diameter, 1–7 cm, and less than 1 cm, for birch, beech, sessile oak, and hornbeam. Particleboards were made from the first thinnings of different types using the proportions of various grades of chips. Similar results were obtained from all the chips made from material greater than 1 cm in diameter. Incorporation of material less than 1 cm diameter greatly reduced the strength and stability of the boards, but acceptable boards could be made if the proportion of this material was reduced to about 5% (from 15–20% in the total biomass), e.g., by using it only in the core layer of three-layer boards. The tree species used turned out to be suitable, as the problem was only with first thinning diameters.

Pedieu et al. [87] proposed to substitute the traditional wood raw material in the surface layers of particleboard with the water-resistant white birch (*Betula papyrifera*) outer bark particles, which helps to improve the dimensional stability of manufactured mixed particleboard and thus reduces the shortages of raw material in a cost-efficient manner. Mixed particleboard was manufactured under laboratory conditions using untreated or alkali-treated white birch outer bark particles as a substitute material. These particles were successively resinated with 3% phenol-formaldehyde resin. Overall, the results of the study demonstrate that the wood-based panels could be manufactured using up to 45% of the proposed substitute material and still maintain the required physical and mechanical properties of particleboard.

#### 3.2.5. Overall Evaluation of the Context of Birch Wood’s Possibilities in Particleboard Production

From the listed and other unmentioned literature sources, birch has excellent prerequisites for its processing into particleboard. Quality assortments of all classes are used for the mechanical processing of warty birch (*Betula verrucosa* Ehrh.) raw wood. To produce birch lumber and blanks, no sawing machine is dominant, and therefore, neither is the cutting technology. Birchwood is well-processed by cutting, which is why we can produce quality lumber with frame saws, log band saws, and log circular saws. Birch dries more slowly and shrivels. Wood tends to vaporize, so sufficient air flow must be ensured during natural drying. Hot-air dryers are mainly used for artificial drying. This wood must be dried more slowly, and the drying temperatures are lower than for conifers. Compared to beech, birch’s drying times are shorter. Birch does not significantly change color during drying. Cuts from manufactured lumber can be found in furniture products, floors, parquets, toy products, and products with useful properties in the home and in restaurant facilities. Birchwood is excellently processed by peeling and cutting. This feature is used in the production of tangential decorative veneers and the structural veneers for the elaboration of all types of plywood panels, including molded plywood and laminated wood. In the Nordic countries, birch is considered the most important wood for plywood. Birchwood in a lower quality class is used for the pulp and paper industry, and also as a welcome raw material in the production of agglomerated materials. The good machinability of birch wood is used in artistic carving. An inherent commercial use of birch wood is its sale as fireplace fuel [28].

Birch wood has proven itself in so many cases in terms of its processing that there are almost no doubts about its incorporation into the core layer of particleboard. It will be necessary to follow the technical principles and reveal the minor imperfections of birch wood by other authors in the past, and the processing of birch wood in some proportion in particleboard will probably be possible.

## 4. Larch Species

### 4.1. Potential of Larch in Terms of its Suitability for Chipping and Fragmentation into Fine Particles

Unlike the two previous trees (alder, birch), European larch and other larch species are softwood (coniferous) species. In our considerations, we also consider it a lesser-known and lesser-used wood species for the purpose of particleboard production, and we also assume that in a certain amount it could be added to the mixture of particles in the middle layer of particleboard, similarly to alder and birch woods, as discussed in Section 2.1 and Section 3.1. Available data on the potential of larch in terms of its suitability for chipping and fragmentation to fine particles were collected. Due to the specifics of this softwood species, some knowledge gained is different from hardwood (alder, birch) species, but it is usable and valuable for us. This knowledge will also be used in the laboratory works and in the preparations for them in such a way that they serve the final goal—to correctly lay out and think about the composition of the middle layers of the particleboard with the possible incorporation of certain proportions of larch wood.

European larch (*Larix decidua* MILL.) has a colored core in the wood. According to the size of the coloration of the core, two basic forms are distinguished. The sapwood can be narrow and yellowish, the core reddish-brown to deep red; at other times, the sapwood is wider and the core lighter. The annual rings are delimited by darker summer wood. Resin channels are scattered in the spring—lighter wood. The wood is valuable for its color and durability. It should be applicable to the particleboard production as a whole, including all the connections resulting from its detailed composition.

The predominant element of the anatomical structure are tracheids—coils that occupy up to 89–93% of the total volume of the wood. They fulfill a conductive and mechanical function. In summer wood, they are thick-walled and tangentially thickened. The cell wall thickness of spring wood is 3.4–5.9–8.2 μm, and of summer wood it is 6.7–8.9–11.4 μm. The length of the tracheids is 2.3–3.4–4.3 mm. The lumen dimensions of spring wood are in the interval of 35.5–43.0–55.0 μm, and summer wood of 4.6–13.0–21.4 μm. The microscopic parameters of larch wood are different than in the case of the hardwoods investigated and presented; in any case, they indicate good possibilities for splitting and chipping the wood.

Larch wood is light, flexible, easy to work with, splitable, and durable even under water.

An overview of the basic physical properties of larch wood is provided in Table 6. An overview of the basic mechanical properties of larch wood at a moisture content of 12% and a moisture content higher than 30% is provided in Table 7. The durability of larch wood in years is listed in Table 8.

Larch can be classified as a very durable tree species in terms of its durability in the air. In terms of its durability in contact with the ground, where there is a risk of damage by all types of rot, it is classified as a medium-durable tree species.

The chemical composition of larch wood is varied by the origin, sampling, age, and state of health. Larch wood contains approximately 50.0% carbon, approximately 42.12% cellulose, 31.95% hemicelluloses, and 26.40% lignin by mass in its absolutely dry state. Larch extractives are typically lipids, phenolic compounds, terpenoids, fatty acids, resin acids, sterols and sterol esters, and waxes. The amount of extractives is always low; in the case of larch, 3.32 [91].

Larch wood was examined in relative detail by many authors, as is evident from the collected values of its physical and mechanical properties from the literature cited. We can therefore assume that these data will be beneficial for the direction of our further research regarding this species. It seems to us that this wood can be very interesting for its use in the central zone of the particleboard, since there is a relationship with spruce, which is currently the main wood used for particleboard production. Section 4.2 provides a summary of the knowledge of various authors about the use of larch wood for particleboard purposes and about the context of larch processing.

### 4.2. Larch in Particleboard and the Context of Its Processing

This subsection also considers the well-known fact that particleboard is at present a necessary, high-quality, highly represented, and very popular engineered wood-based panel composite manufactured from wood elements and synthetic adhesives or other suitable binders. It consists of varying sizes and shapes of particles from lignocellulosic materials, known and tested raw material, but also lesser-known and even non-wood raw material, bonded together with an adhesive under a high temperature and pressure. Particleboard production has positive effects on the environment due to the utilization of various wood residues. The replacement of input raw materials, even if only in a minor volume, brings various technological changes and must be perfectly solved in terms of the quality of the board offered to customers.

The gluing of larch wood is comprehensively addressed, e.g., in the related paper by Chauzov et al. [92]. Their paper presents a study concerning the influence of the density of larch wood on the strength of adhesive joints and its ability in relation to wet modified adhesive. The physical and mechanical properties of adhesive joints are investigated based on a modified melamine-urea-formaldehyde (MUF) adhesive. The application of MUF adhesives allows the creation of glue joints of larch wood with the required strength characteristics. In the formation of adhesive bonds, a sorting of larch by density is necessary. The use of the MUF adhesive composition may reduce the costs of the glue by 26%.

A clear trend in the production of particleboard today is to achieve quality by reducing the input costs associated with the lack of suitable input raw materials and reducing the density of the particleboards produced. Input raw material is not always an incentive for the quality of the particleboard and a meaningful proportion of different input components is decisive for the quality of the particleboard. From this point of view, we also investigate the possible proportion of larch in the central layer of the particleboard so that the necessary furniture quality of the boards is achieved and the unprocessed larch wood is used for an appropriate purpose.

#### 4.2.1. Previous Experience of Particleboard Production from Larch Wood

Pazio and Boruszewski [93] performed an analysis of the influence of larch fibers and particles on selected properties of particleboard and fiberboard. Their paper presents the results of research on the effect of the addition of particles and fibers obtained from European larch wood (*Larix decidua* Mill.) from plantations on selected properties of particleboard and fiberboard in comparison to boards of the same structure based on typical industrial raw material uses by the wood-based panels industry. The differences were proven in the tests of the internal bond, modulus of rupture, modulus of elasticity in static bending, soaking in water, thickness swelling after 2 and 24 h and the density profile. Larch boards with a minimum 50% fiber share were characterized by the comparable values of the properties determined to standard boards regarding the modulus of rupture and modulus of elasticity tests; however, in the remaining variants, pine boards had better properties. The larch boards were evaluated as boards with significantly lower values of swelling by thickness than boards made of wood from forest cultivation. On the other hand, the density profile of the boards on the cross-section of the plantation raw material did not differ from the boards made of pine raw material.

Some authors have examined particleboard using larch in 100% representation, which is not a long-term solution in terms of practice, but it is necessary to become acquainted with the results of their research. Muhcu et al. [94] investigated the log position impact of the European Larch (*Larix decidua* Mill.) tree on the particleboard surface properties as well as the physical and mechanical properties. The logs used by them were split into five segments from the tree butt to the treetop. The wall thickness and fiber length of the wood decreased with the increasing tree height, while the lumen diameter reduced. Likewise, the amount of lignin and cellulose decreased with an increasing tree height, and on the other hand, the number of hemicelluloses increased. The highest values of the solubility (cold and hot water, alcohol-benzene, NaOH) and wood pH were detected in the butt log, continued by the middle log, and finally, the top log. The mechanical properties (internal bond, modulus of rupture, modulus of elasticity) and physical properties (water absorption, thickness swelling), as well as the particleboard surface quality (contact angle and surface roughness), were negatively affected by an increased tree height. The best particleboard properties were obtained for the boards produced from the wood elements of the butt log (0–3 m).

Bardak et al. [95] dealt with the effect of the utilization of sapwood and heartwood on the particleboard surface properties, mechanical and physical properties, and the formaldehyde emissions from particleboard. Trees of European Larch (*Larix decidua* Mill.) were chosen by them as raw materials. The logs were divided into only up to three segments: heartwood, sapwood, and total wood. The authors were interested in the proportions of cellulose. The amounts of it were 51.54%, and that was, of course, the highest number and hemicelluloses (22.24%) in the sapwood, followed by the total wood, and finally, the heartwood. On the other hand, the amount of lignin (30.54%) was found in the heartwood and that was the highest proportion. The extractive values were also surveyed; the highest were obtained from heartwood, followed by total wood, and finally, sapwood. The lowest pH value (3.03) was found in heartwood, while the sapwood samples provided the highest values (4.95). The test samples manufactured from pure sapwood have the smoothest surface and the lowest contact angles; on the other hand, the roughest surface and highest contact angle were acquired from the panel boards of pure heartwood. The formaldehyde emission values and the thickness swelling of the particleboard prepared from pure heartwood were significantly lower than the particleboard prepared from total wood and pure sapwood. The highest internal bond values, and the values of the modulus of rupture and modulus of elasticity, were identified in pure sapwood. The particleboard mechanical strength values are followed by the total wood and the pure heartwood. The particleboard wettability and the surface smoothness manufactured from pure sapwood are higher than those of total wood and pure heartwood.

Lee and Chung [96] assessed larch wood from the point of view of the effect of the temperature and press time on the physical properties of larch particleboard and it appeared to them that larch can be used for making particleboard.

Matsumoto et al. [97] looked at the problem of the effects of the particle size on the properties of particleboard made from larch. They determined the appropriate sizes of the elements, and they concluded that larch is a suitable tree species for making particleboard. This is crucial information if we consider that this research took place at the end of the last century and its results are fully applicable for the intended pressing of larch particles in the mixture in particleboard. We are aware that adhesives and adhesive mixtures for particle pressing are different, more environmentally friendly and less harmful, but the published dimensions of the particles (and fibers) are clearly valuable and workable.

Also, Dix and Roffael [98] have been active in larch research since 40 years ago and they examined the pure larch particleboard from the point of view of the mechanical and technological properties and, in particular, the impact of larch sapwood and heartwood on the particleboard quality. This is all very stimulating information for us.

Shupe et al. [99] determined the effect of five different silvicultural strategies and wood types on the physical and mechanical properties of loblolly pine (*Pinus taeda* L.) particleboard as well as fiberboard. Their entire research is based on comparing their particleboard with particleboard made from larch (*Larix decidua* Mill.). The furnish was prepared unconventionally from veneer outerwood and innerwood for each stand. Regarding the particleboard, the modulus of rupture differences between the stands were insignificant. Certain significant moduli of elasticity differences existed between the stands for fiberboard and particleboard. The differences between the wood types were irrelevant for each stand. For most of the stands, innerwood yielded a higher mean of mechanical values than outerwood. The differences between the stand and wood types for the 2 and 24 h thickness swelling and water adsorption were very small. These trials have shown that innerwood can produce fiberboard as well as particleboard panels with very comparable physical and mechanical properties to outerwood at the level of particleboard made from larch. The effect of the silvicultural strategy (i.e., stand) was almost insignificant for most properties.

Simatupang et al. [100] examined larch, birch, poplar, and oak wood cut in spring when seasoned in the open air. Birch and poplar were processed by chipping after periods of 2, 4, 8, 16, and 32 weeks. One portion of the chips was artificially dried or extracted with water. The other portion was used immediately, and the laboratory boards were elaborated. The researchers used a rather unconventional binder, but their research is nonetheless remarkable. The binders used by them were pure cement or a mixture of cement with condensed silica fume. The boards’ bending strength increased with seasoning time for all the investigated woods but decreased after 32 weeks of seasoning. Artificial drying of the elements and the addition of condensed silica fume led to higher bending strength values. Larch, birch, and poplar wood seasoned for two weeks and oakwood seasoned for two and twelve weeks were used to make composites with different amounts of condensed silica fume (optimum between 25 and 45%). For the usually unsuitable larch and oak wood, the addition of silicon provided boards with good strength values. In their research, it is summarized that nearly all lignocellulosic materials can be used to produce cement-bonded particleboard if condensed silica fume is present.

#### 4.2.2. Inorganic Adhesives Used for the Particleboard Production from Larch

A well-known expert in joining wood with cement, Moslemi [101] considered a full range of particleboard treatments to determine the treatments’ impact on wood–cement cohesion. This was another study of his with a cement binder; however, the species used was larch. His research treatments included hot water extraction of water-soluble components in wood or in chemical additives. The wood species involved included western larch and lodgepole pine. These species turned out to be highly inhibitory in a series of hydration tests. The obtained data presented in the paper show that substantial improvements in cement setting can be achieved by the removal of sugars and water-soluble extractives from western larch. In the case of lodgepole pine, such improvements did not take place. The data present that lodgepole pine has far less of an inhibitory effect than larch in the untreated state. The subsequent addition of chemical additives, especially calcium chloride, appears to enhance compatibility in cement–wood–water mixes. Thus, larch turned out to be a more suitable tree species than pine.

An important study was performed by Simatupang and Geimer [102] with larch wood particles to increase the compatibility of wood-based composites manufactured with inorganic binders of gypsum, Portland cement, or magnesia cement. The result of a series of chemical reactions is the setting of inorganic binders, causing a succession of crystallization stages. Crystallization is inhibited to a diverse degree by various wood kinds and their extractives. The inhibition effect can be reduced by wood aging, and it can be counteracted to certain degree with chemical additives. There have been developed new incorporating processes of solid, liquid, or gaseous additives with the aim of promoting the rapid curing of inorganic bonded boards based on the chemical composition of the wood used. We know from this paper as well as from other papers that larch is an acceptable wood species for these technological operations. The raw material for the binder was obtained as a by-product of other processes in some cases, thus lowering the costs as well. The expansion of inorganic bonded board markets worldwide depends on an increased need for building homes, the availability of materials, as well as on the regulatory codes and changes in established building practices.

Keegan et al. [103] have dealt in detail with larch processing from the point of view of its use in reconstituted board products such as particleboard and medium-density fiberboard. They stated that although there exists information on the use of larch as a raw material to produce pulp and paper, and this is also a good solution, larch as a raw material is a more suitable and economical solution in particleboard or fiberboard products.

#### 4.2.3. Analysis of Chemical Composition of Larch Wood and Bark as a Factor in Particleboard Production

The chemical analyses performed of larch wood or larch bark published in scientific publications seem to be stimulating the research results for incorporating larch particles into particleboards, e.g., Gou et al. [104]. They characterized the nitrogen forms in larch wood particleboard. They used X-ray photoelectron spectroscopy (XPS) in the research to test the existing forms of nitrogen in larch wood, urea-formaldehyde (UF) adhesive, and finally, the particleboard made from larch wood elements and UF adhesive. Their results pointed out that the present nitrogen forms in larch wood are the pyrrolic structures and the amine family, and the nitrogen form in UF adhesive is the amine family only. As expected, the forms of nitrogen in larch wood particleboard and UF adhesive are the amine family and structures, and the percentage of amine nitrogen structures partially increased if compared to the percentage in larch wood. Their results contribute to the research on the release characterization of nitrogen wood species during heating and during wood-based panel pressing.

Liu et al. [105], in their study, investigate the volatile organic compound (VOC) emission of particleboard made of larch under diverse processing conditions. To analyze the VOC components and quantities, they used the equipment of a VOC collection chamber and gas chromatography–mass spectrometer. The emission rate and the concentration of the VOC were substantially affected by the hot-pressing time and temperature. With an increasing hot-pressing time and temperature, both the earlier emission concentration and the amount of total VOC increased. The composition of VOC was also influenced by the time and temperature, mostly the variety of benzene, terpene, and derivative. As for the esters, their existence and quantities were still the main components of VOC emissions.

Lo and Liew [106] divided their study of the Acacia hybrid into bark, sapwood, and heartwood, and they compared their results with other woody species, including larch. Each portion underwent Soxhlet extraction. During the particleboard production, a considerable number of wood elements was extracted using the ratio of extraction of wood particles to solvent extraction. The wood elements were bonded using urea-formaldehyde adhesive and the targeted particleboard density was 500 kg/m^3^. Particleboard made from methanol-extracted wood elements for heartwood and sapwood had a provable difference in mechanical properties compared to the control sapwood particleboard. On the other hand, the heartwood extracted by methanol was higher as compared to the control heartwood. In general, it can be stated that the wood elements extracted by different extraction solvents could be used in particleboard production, but better particleboard properties were proven for methanol-extracted sapwood and heartwood and for hexane-extracted bark. The importance of other wood species for particleboard production is not disputed, and their proposals for achieving higher mechanical properties are probably more complex in terms of the instrumentation and more time-consuming.

Tudor et al. [107] analyzed the possible bark capacity of larch for formaldehyde removal in wood adhesives. Their research activities described the methods of wood-based composite production from larch bark. Five different types of adhesive systems were used (urea-formaldehyde, the mixture of 70% urea-formaldehyde + 30% polyvinyl acetate, polyvinyl acetate, polyurethane, and tannin-based adhesive) and the formaldehyde content of the composites based on larch bark (600 kg/m^3^) was analyzed. A self-agglomerated board was analyzed as well. The findings of the research are that all the tested samples reached the E1 classification (≤8 mg/100 oven dry), and especially in the case of particleboard bonded with tannin-based adhesive, the natural polymer acted as a significant formaldehyde scavenger.

Kain et al. [108], within their study, discussed thermal insulation wood-based panels made from larch, spruce, fir, pine, and oak tree bark with various adhesives (urea-formaldehyde, melamine-formaldehyde, Quebracho, Mimosa) as the wood element binder. The properties of wood-based panels made from larch bark mixed with industrial popcorn were investigated as well. The mechanical and physical properties of the panels were analyzed. They are significantly dependent on the particle size, panel density, bark species, adhesive type, and adhesive content. The bark species has an irrelevant influence on the mechanical properties of the wood-based panels, but the compression ratio is important for the panel strength and stiffness, and therefore, barks with lower bulk density are more preferable. Panels made with green tannin resins under laboratory conditions proved to have adequate properties for practical applications. The addition of a certain amount of popcorn is a relatively suitable means of decreasing the panel density, but the water absorption of such panels shall be comparatively increased. The bark type has a meaningless influence on the thermal conductivity of the wood-based panels; this parameter is predominantly affected by the panel density.

Further scientific research by Tudor et al. [109] aimed at the sound-absorption coefficient of larch bark-based insulation panel products and that is another look at the use of larch wood (and bark) and information for incorporating larch wood into particleboard. The main objective of this study was to investigate the sound absorption coefficient of bark-based insulation panel products made of softwood spruce bark (*Picea abies* (L.) H. Karst.) and larch bark (*Larix decidua* Mill.) with the help of the use of an impedance tube, with its frequency range set between 125 and 4000 Hz. The highest efficiency of sound absorption was noticed for spruce bark-based insulation panels bonded with urea-formaldehyde adhesive, at a level of 1000 and 2000 Hz. The same frequency interval covers the potential for noise reduction of larch bark-based panels glued with tannin-based adhesives. The experimental data show that softwood bark, which is generally considered an underrated material, can substitute for rather expensive materials that involve more gray energy in sound insulation applications. The sound absorption coefficient values therefore strengthen the application of insulation panels based on bark from the forest trees as structural elements for noise reduction in current residential buildings, and they concurrently open up new ways for deeper research in this field.

Kain et al. [110] performed an interesting qualitative investigation on VOC emissions from Norway spruce (*Picea abies*) and larch (*Larix decidua*) loose bark and bark-based panels. Spruce and larch barks were dried first using different methods. Then, the barks were prepared as loose wood elements and panel products. The VOC emissions were measured in a small chamber after 3 and 14 days using gas chromatography and coupled mass spectroscopy. This quantified the influence of the bark type, drying method, and time of hot pressing on the VOC emissions. There were higher total VOC emissions from spruce bark than those from larch bark. High pressing temperature treatments and pressing time significantly decrease emissions from the investigated bark types. In the emitting gases, terpenes, aldehydes, and acids were analyzed within the study.

Liu et al. [111] conducted research on the particleboard mechanical properties and particleboard formaldehyde emissions with nanomaterial-added melamine-impregnated papers, and larch particles were the input raw material for particleboard production. They investigated the influences of the different surface hot-pressing parameters, including the time, temperature, and pressing pressure, on the particleboard properties with nanomaterial-added melamine-impregnated papers. An orthogonal experimental design was employed to investigate the impact of different factors and to determine the optimum levels of the parameters to attain the best desirable board properties. The physical and mechanical properties, including the internal bond strength, modulus of rupture, thickness swelling, and formaldehyde emissions, were determined. With the incorporation of nanoparticles into the adhesives, the physical and mechanical properties of the coated particleboard improved, and the formaldehyde emissions reduced accordingly.

Liu et al. [112] identified and characterized the odorous volatile organic compounds (VOC) emitted from wood-based panel products, and larch elements were in their sights with particleboard production again. VOCs with low concentrations, even below the detection limit, can have the negative effects as well. Therefore, VOCs emitted from particleboard and other laminated boards were precisely collected and analyzed, and the main odorous compounds were identified using the odor activity value. Compared with laminated boards, particleboard demonstrated a higher concentration of total VOCs. Halogenated compounds, esters, and aromatic hydrocarbons were the essential contributors to the total VOCs emissions, which could relate to the additive’s addition when used for panel manufacturing.

Even Bednarczyk and Boruszewski [113] researched larch particleboard, particularly lightweight particleboard, with the manufacturing modification using a blowing agent from the group of bicarbonates. The low-density particleboard has grown in popularity in recent years due to its facilitated transportation and the lower own mass in the assembled finished products. However, there are still certain restrictions in the general utilization caused by the insufficient, sometimes sorely lacking, mechanical properties. These deficiencies may be controlled by the mechanism of foaming the polymers that bind wood particles in the structure of the boards. The study aimed to determine the possibility of using sodium bicarbonate as a blowing agent of phenolic resin used for bonding wood particles in the technology of lightweight particleboard. It was found that the addition of sodium bicarbonate in the amount of 5% of the dry weight of the phenolic resin substantially increased the internal bond strength of ready-made the particleboard.

The idea of adhesives-free bark panels is also interesting and is addressed in some scientific works, e.g., Wenig et al. [114]. They engaged with adhesives-free bark panels as an alternative application for an accumulated waste material. The motivation for this research was to conserve the bark in a natural common state and to explore alternative processes and applications for its utilization. The common method of tree trunk debarking by peeling was used to obtain, if possible, large bark pieces. The procedure was that two pieces of peeled bark were placed crosswise, approximately with the rhytidome sides (outer bark) facing each other. After diverse conditioning conditions, the bark pieces were pressed with the help of a high temperature to panel products without using any adhesives. The experiment on bark samples of various Central European tree species (including larch) revealed that panel product production with species-dependent properties is basically possible and feasible with certain changes in technology. These are the steps toward the production of the sustainable panel products by the incorporation of the natural waste material, and at the same time, to keep its beneficial structure and its naturally real chemical composition.

Kajita and Imamura [115] carried out a study with chemically modified particleboard and fiberboard made from larch (*Larix leptolepis* G.), mostly to improve the mechanical properties, but also the dimensional stability and biological properties, too. The upgraded technology developed by the authors from the solid wood chemical modification was applied to these targeted reconstituted wood products. Their research report described the means of production and the identified properties of the chemically modified panel boards.

It seems that nowadays larch bark has been more discussed in the research than larch wood. An example is the scientific work by Ninikas et al. [116]. They examined the technical sensuality of low-density insulation particleboard manufacturing. Particleboards were produced from two renewable resources, first tree bark and second hemp fibers (*Cannabis sativa*). Non-toxic methyl cellulose glue was used by them as a binder. Four types of panel products were made, which corresponded to varying mixtures of tree bark (including larch particles) and hemp fibers (tree bark to hemp fibers per-centages of 90:10, 80:20, 70:30, and 60:40). An additional set of panels was produced, consisting only of tree bark as a comparative material. The results achieved pointed out that the hemp fiber addition partially improved the mechanical properties of the boards and an acceptable level of the boards was achieved. However, a negative side effect was that the thermal conductivity increased as the hemp content increased. However, it was gratifying that all the values were still within the acceptable range. Board type 30:70 (with hemp content of 30%) had the highest mechanical properties as well as the optimal thermal conductivity value based on the cluster analysis. So, based on this research, it was concluded that insulation panels with a low density can be successfully produced using these renewable waste raw materials.

The work by Oktoberyani et al. [117] is also worth noting. The authors fabricated the bark particleboard without binders from gelam (*Melaleuca viridiflora* Sol. ex Gaertn.) and other woody species, including larch. They looked at the issue in terms of bark as a waste product. They investigated the influence of the pressing temperature on the particleboard’s physical and mechanical properties. The thermal insulation particleboard properties were determined by them as well. Four different temperatures (140 °C, 160 °C, 180 °C, and 200 °C) were used, with the aim to laboratory-produce single-layer binderless bark particleboard with a target density of less than 590 kg/m^3^. Their results showed that the pressing temperature, of course, affected the mechanical properties (tensile strength perpendicular to panel surface, modulus of rupture, and modulus of elasticity) and these mechanical properties increased as the pressing temperature increased. The particleboard’s physical properties (water absorption and thickness swelling) decreased as the pressing temperature increased. However, the increase in the pressing temperature from 180 °C by 20 °C more did not affect the particleboard’s physical or mechanical properties significantly, except for the tensile strength perpendicular to the panel surface. Another positive was that the binderless bark particleboard that was hot-pressed at 200 °C had high water resistance, regardless of its low strength. Its thermal conductivity value was 0.14 W/m·K.

The study by Czajka and Fabisiak [118] determined the radial variation in the content of cellulose and lignin in the cross-section of the chosen softwood species, including larch (*Larix decidua* Mill.). The average values of the content of cellulose and lignin calculated for the entire cross-sections of the larch wood were very similar. In the case of cellulose, for larch wood its average content was 48.29%, and the average content of lignin was 27.93%. Lower values of the cellulose share and higher of the lignin share were obtained for the juvenile larch wood zone compared to the mature larch wood zone. 

#### 4.2.4. Overall Evaluation of the Context of Larch Wood’s Possibilities in Particleboard Production

It is clear from the mentioned and other unmentioned literature sources that larch wood particles and larch bark particles are suitable prerequisites for their processing into particleboard. Assortments of raw larch wood (*Larix decidua* MILL.) are intended for mechanical processing by sawing technologies as well. Larch is easy to use in this way. Like other types of softwoods, larch cutouts are transformed into lumber by cutting, primarily with frame saws. The mechanical as well as the physical properties of larch wood, but above all its unique color and texture, predetermine it for the production of all products, sometimes more than the industrially more important spruce wood. Its more limited application is due to its lower occurrence in forests compared to spruce. Larch lumber is highly valued in construction and carpentry products, garden architecture products, and exterior and interior floors and coverings. Of course, it achieves the highest market value in the production of all kinds of rustic furniture—sofas, tables, cabinets, and beds. The highest quality assortments of larch wood are used in shipbuilding for structural but mainly decorative purposes. Larch wood dries more slowly than other conifers, and it tends to slightly warp and crack. During natural drying, it is stored with smaller gaps between the sides of the lumber, and thick lintels are not used. Smaller distances are left between the cages, which shade them from the sun. The cages are loaded and protected against rain. For artificial drying, hot-air dryers are mainly used, but also vacuum and condensation dryers. During drying, there is no significant change in the color of the wood. An accompanying phenomenon is the oozing of resin and the release of non-healed knots. For decorative purposes, larch wood is processed in veneer production with cutting machines. These are the highest-quality cutouts. Quality assortments of larch raw wood of a slightly lower quality class are the raw materials for the production of structural veneers or plywood panels. Larch wood of a low quality class is used for the pulp and paper industry, as well as for the production of agglomerated boards, for the production of packaging, and pallets, and for the production of toys, jewelry, and beekeeping supplies. Sometimes larch is mistakenly used in products as an alternative to spruce, fir, and pine wood, but as this literature review clearly shows, this is not correct. Larch has valuable properties and should be considered a first-class and full-fledged woody species [28].

From this collected theoretical information, it appears that larch wood will be usable for particleboard production and for bonding the particles in a mixture with spruce particles into the core layers of particleboard. Although there is less information than for birch wood, in the context of using a mixture of two coniferous species with rather similar chemical compositions (larch and spruce), this board-forming process is probably feasible. In terms of the proportion of larch wood shares in the particleboard core layer (similar to the alder wood and birch wood described in Section 2 and Section 3), this will be the subject of future research activities, with which all this collected information will assist.

The latest publication by Akbulut and Ayrılmış [119] confirmed the current optimal technologies for particle production from massive wood and confirmed the optimal procedures for gluing particles into particleboard as well. The information contained in it is also an assumption that modern technologies used for particleboard production are suitable for processing lesser-known and lesser-used wood species, and that no additional investments are required. Wood, as a raw material for particleboard production in 100% representation, will always be the preferred option compared to alternative shares of non-wood raw materials.

## 5. Conclusions

Lesser-known European species of alder, larch, and birch may be used as additives to major input raw material in the particleboard core layer. This literature search confirmed the diversity of use of these three trees in different fields and unequivocally showed their adequate physical and mechanical properties and chemical composition as usable tree species to cover part of the raw material needs for particleboard.

Of course, the greatest experience in use is with Nordic varieties of birch, which is a very valuable raw material in Northern Europe. However, birch is beginning to gain ground in other parts of the world, and the analysis of its wood and chips in the cited works confirmed this. Birch is also likely to prove useful in the experiments we intend to perform as part of the upcoming research.

Larch is currently a highly researched tree species and not only larch wood but also larch bark has great potential. In particular, the use of larch bark in composites is researched at several research institutes around the world, and the properties of larch composites are tested and refined by various procedures, including experimental ones, to achieve excellent results.

Alder wood has such a beautiful and original color that it is predestined for the manufacture of sliced veneers. Of course, in this case, they must be trees with certain meaningful diameters so that sliced veneers with the necessary dimensions are produced. Smaller diameters of alder wood are very suitable for making particleboard in terms of their chemical and technical properties and their incorporation into particleboard is confirmed by this literature review.

These three tree species provide an eco-friendly and sustainable alternative to the conventional tree species used in the wood-based panel industry. They cannot be a full-fledged replacement, given that their occurrence in forests and wood reserves is not very high, but they can reduce the spruce raw material in particleboard, which is less accessible for the composite industry and there exists a great demand for it in other industrial areas. However, in this respect, fundamental research is still needed to determine the proportional shares of use of these tree species in particleboard for formulating the correct technology shares and rules for their application in the wood-based panel industry. This literature survey confirmed that the potential of alder, birch, and larch for use in the center layer of particleboard is considerable.

## Figures and Tables

**Table 1:** Overview of the basic physical properties of alder wood [29,30,32,35].

Density (kg/m^3^)		The Moisture Content of Freshly Cut Wood (%)		Fiber Saturation Point (FSP) (%)
In Absolute Dry State ρ_o_	Moisture Content of 30% ρ_rf_	Sapwood	Mature Wood	
450–510–600	530	80–110		31–34
Shrinkage (%)	Longitudinal	Radial	Tangential	Volumetric
	0.5	4.6	8.5	13.8

**Table 2:** Overview of the basic mechanical properties of alder wood at a moisture content of 12% [29,32,35,36].

Property	Parallel to the Fibers w = 12%	Perpendicular to the Fibers w = 12%
Tensile strength (MPa)	94	7.3
Compressive strength (MPa)	55	
Shear strength (MPa)	4.5	
Bending strength (MPa)	97	
Modulus of elasticity in bending (MPa)	11,700	
Brinell hardness (MPa)	36	14

**Table 3:** Overview of the basic physical properties of birch wood [30,62,63,64].

Density (kg/m^3^)		The Moisture Content of Freshly Cut Wood (%)		Fiber Saturation Point (FSP) (%)
In Absolute Dry State ρ_o_	Moisture Content of 30% ρ_rf_	Sapwood	Mature Wood	
460–800	520	80–90		30–35
Shrinkage (%)	Longitudinal	Radial	Tangential	Volumetric
	0.6	5.3	7.8	14.2

**Table 4:** Overview of the basic mechanical properties of birch wood at a moisture content of 12% and a moisture content higher than 30% [30,62,63,64].

Property	Parallel to the Fibers		Perpendicular to the Fibers	
	w = 12%	w > 30%	w = 12%	w > 30%
Tensile strength (MPa)	137		7.0	4.0
Compressive strength (MPa)	59.9	26.3		
Shear strength (MPa)	16.2	7.7		
Bending strength (MPa)	123	63		
Modulus of elasticity in tension (MPa)	18,100		600	
Modulus of rigidity (MPa)	15,800		600	
Shear elastic modulus (MPa)	1450		800	
Modulus of elasticity in bending (MPa)	13,300	9900	800	
Toughness (J·cm^−2^)			9.32	7.85
Brinell hardness (MPa)	48		20	
Janka hardness (MPa)			40	

**Table 5:** The durability of birch wood in years [28].

Exposure	Unprotected and Unimpregnated	Under the Roof	Under the Water	Always Dry
Durability (years)	3–8–15	5–20–30	20–40–60	300–500

**Table 6:** Overview of the basic physical properties of larch wood [30,88,89,90].

Density (kg/m^3^)		The Moisture Content of Freshly Cut Wood (%)		Fiber Saturation Point (FSP) (%)
In Absolute Dry State ρ_o_	Moisture Content of 30% ρ_rf_	Sapwood	Mature Wood	
400–550–820	460	100	30–40	23–28
Shrinkage (%)	Longitudinal	Radial	Tangential	Volumetric
	0.3	4.3	10.4	15.0

**Table 7:** Overview of the basic mechanical properties of larch wood at a moisture content of 12% and a moisture content higher than 30% [29,32,35,36].

Property	Parallel to the Fibers		Perpendicular to the Fibers	
	w = 12%	w > 30%	w = 12%	w > 30%
Tensile strength (MPa)	107			
Compressive strength (MPa)	55.0	41.0	9.7	
Shear strength (MPa)	12.4	6.9		
Bending strength (MPa)	92	64		
Modulus of elasticity in tension (MPa)	14,500			
Modulus of rigidity (MPa)	14,000		600	
Modulus of elasticity in bending (MPa)	13,800	6300		
Toughness (J·cm^−2^)			6.0	4.0
Brinell hardness (MPa)	53		19	
Janka hardness (MPa)	38		36.5	24.5

**Table 8:** The durability of larch wood in years [28].

Exposure	Unprotected, Unimpregnated, and Impregnated	Under the Roof	Under the Water	Always Dry
Durability (years)	20–60–80	100–120–150	300–500–700	800–1000
